# Ursolic acid alleviates liver injury in diabetic mice induced by high-fat diet combined with streptozotocin via the NLRP3 signaling pathway

**DOI:** 10.1371/journal.pone.0340643

**Published:** 2026-02-04

**Authors:** Xinyan Wang, Ruyu Ma, Di Lou, Hanbing Li, Minyou Qi

**Affiliations:** Institution of pharmacology, Zhejiang University of Technology, Hangzhou, Zhejiang, China; Noorda College of Osteopathic Medicine, UNITED STATES OF AMERICA

## Abstract

Non-alcoholic fatty liver disease (NAFLD) is a type of metabolic stress-induced liver injury that is closely related to type 2 diabetes mellitus (T2DM). Ursolic acid (UA), a natural pentacyclic triterpenoid compound, has anti-inflammatory, hypoglycemic and liver-protective effects. However, its role in regulating liver injury through the NLRP3 inflammasome pathway in a T2DM combined with NAFLD model has not been systematically elucidated. This study systematically evaluated the protective effect of UA on NAFLD and its molecular mechanism through in vivo (STZ + high-fat diet-induced NAFLD mouse model) and in vitro (high glucose + palmitic acid-induced LO2 cell oxidative stress model) experiments. The results showed that UA significantly improved hepatic lipid deposition, reduced serum ALT/AST levels, and effectively alleviated oxidative stress, as indicated by decreased malondialdehyde (MDA) content and increased activities of superoxide dismutase (SOD), catalase (CAT), and glutathione peroxidase (GSH-Px) in liver tissues. Further mechanism studies revealed that UA could significantly down-regulate the expression levels of pro-inflammatory factor IL-1β and pro-fibrotic factor TGF-β1 by inhibiting NLRP3 inflammasome activation, and simultaneously reduce the deposition of type IV collagen. This study demonstrated that ursolic acid (UA) has a protective effect on T2DM combined with NAFLD, and its mechanism of action may be related to the regulation of the NLRP3 signaling pathway by UA, which inhibits oxidative stress, inflammation and fibrosis.

## 1. Introduction

Non-alcoholic fatty liver disease (NAFLD) represents a hepatic manifestation of metabolic syndrome (MS), pathophysiologically linked to core metabolic derangements—visceral adiposity, insulin resistance, type 2 diabetes mellitus (T2DM), hypertension, and dyslipidemia—while concurrently elevating risks for extrahepatic complications including coronary artery disease, chronic kidney disease, sarcopenia, and non-hepatic malignancies. [1]. Indeed, the prevalence of NAFLD is 38% in the general population and 70% in patients with T2DM [2]. It is evident that there is a correlation between the progression of NAFLD and T2DM. As NAFLD progresses, it can affect not only the liver but also other areas of the body, such as the kidneys, cardiovascular system, and respiratory system. This can increase the risk of developing chronic kidney disease, cardiovascular disease, sleep apnoea, type 2 diabetes, and certain cancers [3]. On the other hand, metabolic disorders such as obesity, insulin resistance, hypertension, and hyperlipidaemia can also contribute to the development of NAFLD and non-alcoholic steatohepatitis (NASH) [4]. However, effective treatments for T2DM with NAFLD are still lacking, so the development of a drug to improve this condition is necessary.

The development and progression of T2DM combined with NAFLD are closely associated with oxidative stress and inflammation [5,6]. Persistent hyperglycemia promotes the generation of excessive reactive oxygen species (ROS), leading to liver injury [7]. Meanwhile, overproduction of ROS can activate the NLRP3 inflammasome and trigger inflammatory responses [8]. Studies have indicated that NLRP3 is a key factor in the aggravation of NAFLD [9]. Furthermore, oxidative stress and inflammation can activate hepatic stellate cells (HSCs), which in turn produce extracellular matrix (ECM) proteins and act as central drivers of liver fibrosis [10]. Therefore, modulating oxidative stress, inflammation, and fibrosis through the NLRP3 pathway may represent a core mechanism for improving T2DM combined with NAFLD.

**Fig 1:**
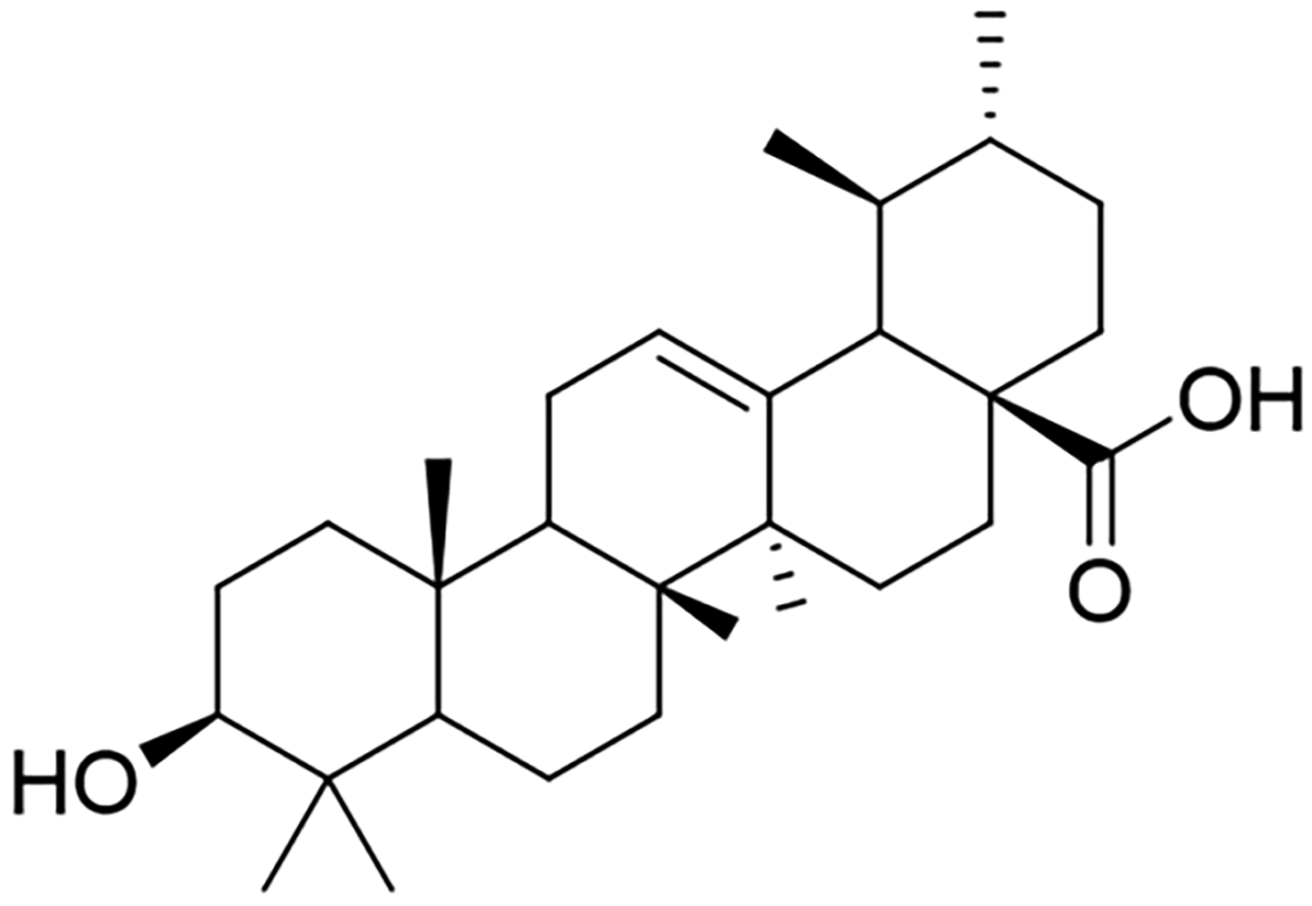
The chemical structure of UA.

Ursolic acid (UA) (Fig 1) is a pentacyclic triterpenoid compound widely distributed in the flowers, buds, and leaves of various medicinal plants, fruits, and vegetables [11–13]. It has been demonstrated to possess remarkable pharmacodynamic properties, exhibiting multiple bioactivities and a high safety profile. It can be stored and accumulated in various organs, including the liver, kidneys, heart, and brain tissues, and has been shown to have a strong potential for the prevention and treatment of a wide range of diseases, including metabolic disorders and obesity, cardiovascular diseases, cancers, and neurological disorders [14,15]. Notably, in the food industry, UA has been utilized as a natural antioxidant and can be developed into functional health foods [16]. Previous studies have indicated that UA can reverse liver fibrosis by inhibiting the NOX4/ROS and RhoA/ROCK1 signaling pathways [17]. In summary, these findings lead us to hypothesize that UA ameliorates diabetes-induced liver injury.

Therefore, the objective of this study is to establish a model of T2DM combined with NAFLD in mice induced by high-fat diet (HFD) and streptozotocin (STZ), as well as a preliminary exploratory LO2 cell model induced by high glucose (HG) and palmitic acid (PA). This will allow for the study of the mechanism by which UA exerts a protective effect on T2DM combined with NAFLD, and to provide a basis and reference for expanding the clinical application of UA.

## 2. Materials and methods

### 2.1. Chemicals and reagents

This study utilized the following reagents: UA (HPLC > 98%, Shanghai Huzhen, China); PA (HPLC > 99%, 57-10-3, Sigma, USA); STZ (HPLC > 99%, 18883-66-4, Sigma, USA); NSS (Casmart, China); fetal bovine serum (FBS) (11011–8611, Tianhang, China); DMEM low-glucose medium (11-885-084, Gibco, USA); aminotransferase (AST) assay kit (C010-2–1, Nanjing Jiancheng, China); alanine aminotransferase (ALT) assay kit (C009-2–1, Nanjing Jiancheng, China); triglyceride (TG) assay kit (A110-1–1, Nanjing Jiancheng, China); total cholesterol (TC) assay kit (A111-1–1, Nanjing Jiancheng, China); superoxide dismutase (SOD) assay kit (A001-3–2, Nanjing Jiancheng, China); catalase (CAT) assay kit (A007-1–1, Nanjing Jiancheng, China); glutathione peroxidase (GSH-Px) assay kit (A006-2–1, Nanjing Jiancheng, China); malondialdehyde(MDA) assay kit (A003-1–2, Nanjing Jiancheng, China); BCA protein concentration assay kit (P0012, Beyotime, China); 3-[4,5-dimethylthiazol-2-yl]-2,5-diphenyltetrazolium bromide (MTT) (ST316, Beyotime, China).

The antibodies used are as follows: NLRP3 Antibody (DF15549, Affinity, China); IL-1β antibody (BF8021, Affinity, China); TGF-βl antibody (BF8012, Affinity, China); Collagen IV antibody (ab6586, Abcam, China); GAPDH antibody (ab181602, Abcam, China). The secondary antibodies used in this study were as follows: goat anti-Rabbit IgG (H + L) antibody (32460, Thermo Scientific, China); goat anti-Mouse IgG (H + L) antibody (A16066, Thermo Scientific, China).

### 2.2. Animals and drug treatment

The male C57BL/6J mice with 7 weeks of age and 20 ± 2 g of body weight were purchased from Hangzhou Ziyuan Experimental Animal Technology Co., Ltd. (Hangzhou, China). All experimental procedures were conducted in accordance with the Guide for the Care and Use of Laboratory Animals in the Zhejiang University of Technology, Hangzhou, China, and conformed to the National Institutes of Health Guide for Care and Use of Laboratory Animals (Publication No. 85−23, revised 1996). The protocol was approved by the Research Ethics Committee of Zhejiang University of Technology (Protocol Number: 20190301014). Every effort was made to minimize the number and suffering of mice included in this study.

Following 7-day acclimatization in specific pathogen-free (SPF) facilities (25 ± 2°C, 50 ± 10% humidity, 12/12 light cycle), C57BL/6J mice were randomized into two cohorts: normal chow group and HFD group. After 6 weeks of dietary intervention, diabetic induction was achieved via daily intraperitoneal injection of streptozotocin (35 mg/kg in 0.1 M citrate buffer, pH 4.5) for 5 consecutive days. Nine days post-STZ administration, fasting blood glucose (FBG) levels were quantified. The mice with a FBG level of ≥11.1 mmol/L were considered diabetic and randomly divided into three groups (n = 8), the normal group (NC), the model group (DC) and the UA group. Mice in the UA group were treated with 100 mg/kg UA(dissolved in saline) by gavage for 8 consecutive weeks [18], while mice in the NC and DC groups were given the same volume of saline as that in the UA group. During the experimental period, mice in the NC group were fed a normal diet, while those in the DC and UA groups were fed a high-fat diet. After the final administration, all mice were fasted for 12 hours with free access to water. Following anesthesia with isoflurane, blood was collected from the tail vein for blood glucose measurement. Subsequently, the mice were anesthetized by intraperitoneal injection of sodium pentobarbital (50 mg/kg) and euthanized by cervical dislocation. All surgical and terminal procedures were performed under adequate anesthesia to ensure that animals did not experience pain or distress. The obtained serum was stored at −20°C for biochemical analysis. A portion of the liver tissue was fixed in 4% paraformaldehyde solution for 24 hours, while the remaining liver tissues were preserved at −80°C for subsequent studies.

### 2.3. Serum and liver tissue biochemical analysis

Prior to euthanasia, fasting blood glucose levels were quantified via tail vein sampling using a calibrated glucometer. Orbital sinus-derived blood samples were allowed to clot for 6 hours at room temperature followed by centrifugation (3,500 × g for 10 min at 4°C) to obtain serum. Hepatic injury biomarkers (ALT and AST) and lipid profiles (TG and TC) were analyzed using commercial enzymatic assay kits (Jiancheng Bioengineering Institute, Nanjing, China) following manufacturer protocols. Excised liver tissues were immediately rinsed with ice-cold saline, gently blotted on sterile absorbent paper, and weighed to calculate liver coefficient using the formula (Liver coefficient (%) = Liver weight (g)/body weight (g) ×100%). A 10% (w/v) homogenate was prepared at a 1:9 ratio. The homogenate underwent centrifugation (1000 × g, 10 min, 4°C) to obtain clarified supernatant, which was aliquoted and stored at −80°C for subsequent oxidative stress analyses. Hepatic oxidative stress parameters (SOD, CAT, GSH-Px and MDA) were quantitatively assessed using validated commercial assay kits (Jiancheng Bioengineering Institute, Nanjing, China).

### 2.4. Histopathological examination

The mice liver tissues were placed in fixative for 24 hours, then dehydrated and subsequently subjected to dip-wax embedding. The samples were then cooled at −20°C and made into wax slices of liver tissues with a thickness of 4μm. The histological examination was conducted using hematoxylin and eosin (H&E) staining, Masson’s trichrome staining, and Oil Red O staining. The resulting images were captured using a light microscope (TI-S, Nikon, Japan). The cells in each group were fixed with 4% paraformaldehyde and stained with freshly configured Oil Red O. Following this, 75% ethanol was added to differentiate for two seconds in order to wash away the residual staining solution. The ethanol was then discarded, and the cells were placed under the microscope for observation.

### 2.5. Immunohistochemical analysis

Following dewaxing and hydration of paraffin sections of liver tissue, antigen repair was performed with EDTA antigen repair buffer. The sections were subjected to an initial 25-minute incubation with a 3% H₂O₂ solution (H₂O₂:H₂O = 1:9) at room temperature under conditions that ensured protection from light. This was followed by a 30-minute closure with 3% BSA solution at room temperature. Thereafter, the sections were sequentially incubated with NLRP3 (1:1000), IL-1β (1:500), TGF-β1 (1:1000), Collagen IV (1:2000) and GAPDH (1:10000) primary antibodies overnight at 4°C. The slices were then rinsed with PBS and incubated with goat anti-rabbit and goat anti-mouse IgG secondary antibodies (Thermo Pierce). Subsequently, the chromogen diaminobenzidine (DAB) was added, and the nuclei were stained with hematoxylin. Following the processes of dehydration and sealing, the images were subjected to microscopic observation and documentation.

### 2.6. Cell culture and treatment

LO2 cells (Wuhan Pricella Biotechnology Co., Ltd., Wuhan, China) were cultured in DMEM low-glucose medium with 10% FBS in an incubator at 37°C with 5% CO_2_. The cells were cultured until they reached approximately 75% fusion and then incubated for 12 hours in serum-free medium. The cells were divided into normal, mannitol, model and UA groups. The normal group and the mannitol group were cultured with DMEM complete medium (5.6 mmol/L glucose) and the same concentration of mannitol hypertonic complete medium respectively. The cells in the model group were treated with DMEM medium containing 0.25 mmol/L palmitic acid and 33.3 mmol/L glucose for 24 hours to induce hepatocyte steatosis. The UA group treated model cells with medium containing 25 μmol/L UA for 24 hours. An additional NLRP3 agonist group was established, including model group, UA group, UA + NSS group, and NSS group.

### 2.7. Cell viability assay

LO2 cells in the logarithmic growth phase were diluted and inoculated into 96-well plates. Each well contained about 5 × 10^3^ cells with a volume of 100 μL. The cells were cultured in an incubator for 24 hours. After removing the old medium, DMEM low-glucose medium (without FBS) was added, and the cells were starved and cultured for 12 hours. After removing the supernatant, normal LO2 cells and LO2 cells induced with steatosis by 0.25 mmol/L palmitic acid and 33.3 mmol/L glucose were treated with different concentrations of UA (6.25, 12.5, 25, 50, 100 μmol/L) for 24 hours. Following treatment, 20 μL of MTT solution (5 mg/L) was added to each well and incubated for 4 hours. The supernatant was then discarded, and DMSO (150 μL/well) was added. The plate was shaken for 10 minutes, and A_570 nm_ was measured.

### 2.8. Cell supernatant biochemical analysis

The cells in each group were collected using a cell scraper under ice water bath conditions and centrifuged into a centrifuge tube (4°C, 1000 rpm/min, 10 min). The supernatant was discarded, and an appropriate amount of RIPA lysate containing 1% Triton X-100 was added to lyse the cells for 30–40 minutes. After lysis, the protein concentration was determined using BCA. The levels of TC, TG, MDA and SOD activity in the cell supernatant were determined by the kit.

### 2.9. Light microscopy

The cells in each group were fixed with 4% paraformaldehyde and stained with freshly prepared oil red O. To differentiate, 75% ethanol was added for 2 seconds and then washed away. The cells were observed under a microscope (TI-S, Nikon, Japan).

### 2.10. Immunofluorescence staining

LO2 cells were fixed in 4% paraformaldehyde for 30 minutes, after which they were washed with PBS and blocked with 10% normal donkey serum for 30 minutes. Then the cells were incubated with the respective antibodies, namely NLRP3, IL-1β, TGF-β1, and Collagen IV, overnight at 4°C. Thereafter, the cells were incubated with the appropriate Alexa Fluor 488 secondary antibody for 60 minutes at room temperature, protected from light. Finally, the nuclei were stained with DAPI and photographed using a laser scanning confocal microscope (LSM, Zeiss, Germany) for photography.

### 2.11. Western blotting

The cells were lysed using Ripa lysate containing 1% TritonX-100 for 30−40 minutes before determining the protein concentration. 0.1 g of liver tissue was homogenised on ice with 990 μL of pre-cooled RIPA lysate and 10 μL of PMSF. The homogenisation was performed for 60 seconds at 60 HZ. After centrifugation (1000 g, 10 min, 4°C), the supernatant was collected and the protein concentration was determined using a BCA kit (Beyotime). Subsequently, the distinct protein samples were subjected to sodium dodecyl sulfate polyacrylamide gel electrophoresis (SDS-PAGE) and transferred onto polyvinylidene difluoride (PVDF) membranes. The membrane blots were then incubated with the corresponding primary antibodies. The following primary antibodies were used: NLRP3 (15101; dilution, 1:1000; CST), IL-1β (ab254360; dilution, 1:500; Abcam), TGF-β1 (ab179695; dilution, 1:1000; Abcam), Collagen IV (50273; dilution, 1:2000; CST) and GAPDH (ab181602; dilution, 1:10000; Abcam). The next day, the PVDF membranes were incubated with goat anti-rabbit and goat anti-mouse IgG secondary antibodies (Thermo Pierce). The protein bands were visualised by enhanced chemiluminescence (ECL), and the optical density values were analysed by Image J.

### 2.12. Statistical analysis

The data were analysed using GraphPad Prism 6.0 and presented as mean ± SD. Multiple comparisons were performed using one-way ANOVA followed by Tukey’s test. A significance level of *P* < 0.05 was considered statistically significant.

## 3. Results

### 3.1. Effects of UA administration on liver injury of T2DM mice

**Fig 2:**
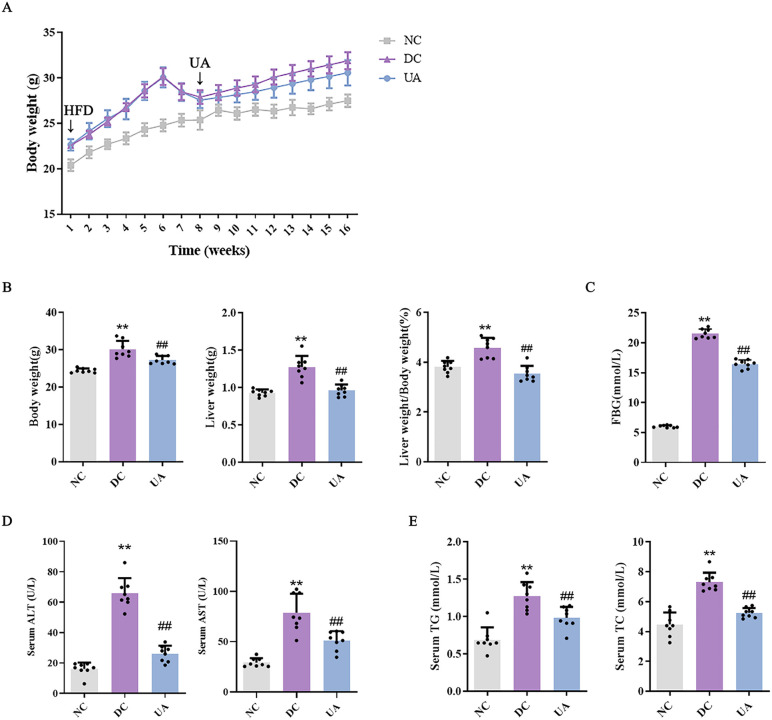
Effects of UA administration on liver injury of T2DM mice. **(A)** Effect of UA on body weight in mice. The HFD started at week 0, and UA administration began at week 8. **(B)** Effect of UA on liver coefficient of mice. **(C)** Effect of UA on fasting blood glucose of mice. **(D)** Serum ALT and AST levels of mice in each group. **(E)** Serum TG and TC levels of mice in each group. ^*##*^*P* < 0.01 versus DC group; ^**^P < 0.01 versus NC group. The data were presented using means ± SD (n = 8).

To further investigate the therapeutic effects of UA on T2DM-associated NAFLD, we established a hepatic injury model in C57BL/6J mice through HFD combined with low-dose streptozotocin. UA treatment significantly ameliorated pathophysiological manifestations in diabetic mice, including lethargy, greasy fur, and delayed responsiveness, and showed a non-significant trend toward reduced body weight gain compared to the DC group (Fig 2A). Quantitative analysis revealed a marked reduction in liver coefficient for UA-treated mice (*P* < 0.01) (Fig 2B). Furthermore, UA administration substantially decreased fasting blood glucose levels (*P* < 0.001) (Fig 2C).

Serum ALT and AST, critical biomarkers of hepatic function, were significantly reduced in the UA-treated cohort compared to the DC group (*P* < 0.01) (Fig 2D), demonstrating UA’s hepatoprotective efficacy in T2DM-NAFLD comorbidity. Lipid profiling revealed UA attenuated dyslipidemia, with serum TG and TC levels decreasing markedly (*P* < 0.01) (Fig 2E).

### 3.2. UA ameliorated hepatic steatosis in histopathology of T2DM mice

**Fig 3:**
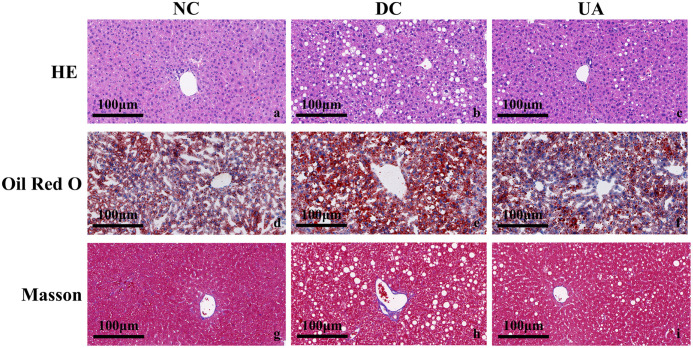
Effects of UA on hepatic histopathological alterations in the HFD/STZ-induced mice. **(a-c)** H&E staining of liver tissue, **(d-f)** Oil Red O staining of liver tissue, **(g-i)** MASSON staining of liver tissue (200×).

As shown in the results, hematoxylin-eosin (HE) staining demonstrated that the liver tissue of mice in the NC group exhibited a clearly delineated contour, normal hepatic sinusoids, radial arrangement of hepatic cords, and an absence of significant fatty degeneration of hepatocytes. In contrast, the structure of hepatic sinusoids in mice in the DC group was compromised, the morphology of hepatocytes was altered, the arrangement of hepatocytes was noticeably disorganized, and a substantial number of fat vacuoles were present within the cytoplasm of the cells. The presence of fat droplets in the hepatic tissues of the DC group was discernible when compared with the NC group, as evidenced by oil-red staining. Furthermore, Masson staining revealed a substantial augmentation in collagen fibers within the liver tissue of DC group mice. Following treatment with UA, a significant reduction in hepatic lipid deposition was observed in mice from the UA group. Additionally, a substantial enhancement in both steatosis and collagen proliferation was noted (Fig 3).

### 3.3. UA alleviates liver oxidative stress in T2DM mice

**Fig 4:**
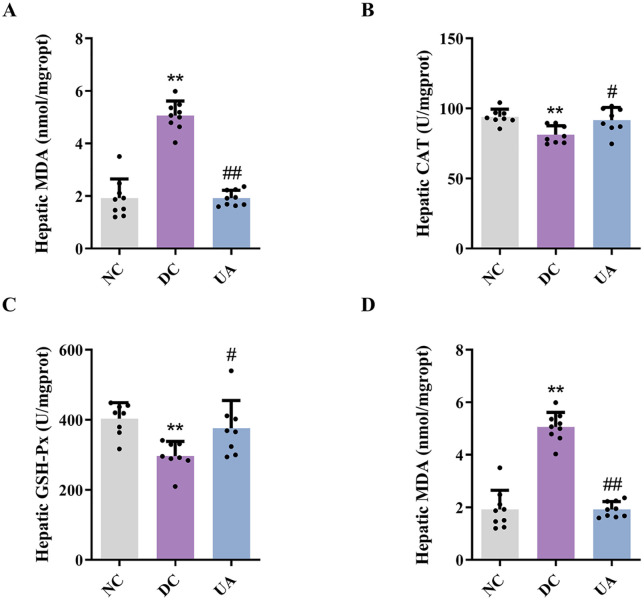
UA alleviates liver oxidative stress in T2DM mice. **(A)** The liver SOD levels of T2DM mice. **(B)** The liver CAT levels of T2DM mice. **(C)** The liver GSH-PX levels of T2DM mice. **(D)** The liver MDA levels of T2DM mice. ^*#*^*P* < 0.05, ^*##*^*P* < 0.01 versus DC group; ^**^P < 0.01 versus NC group. The data were presented using means ± SD (n = 8).

The antioxidant enzymes SOD, CAT, GSH-Px, and MDA have been identified as significant indicators of oxidative stress levels in murine models. The results (Fig 4) showed that UA significantly increased (*P* < 0.05) the levels of SOD, CAT and GSH-Px and significantly decreased (*P* < 0.01) the MDA content in liver tissues of mice, which inhibited the oxidative stress in the livers of mice with T2DM.

### 3.4. UA alleviates liver inflammation and fibrosis in T2DM mice

**Fig 5:**
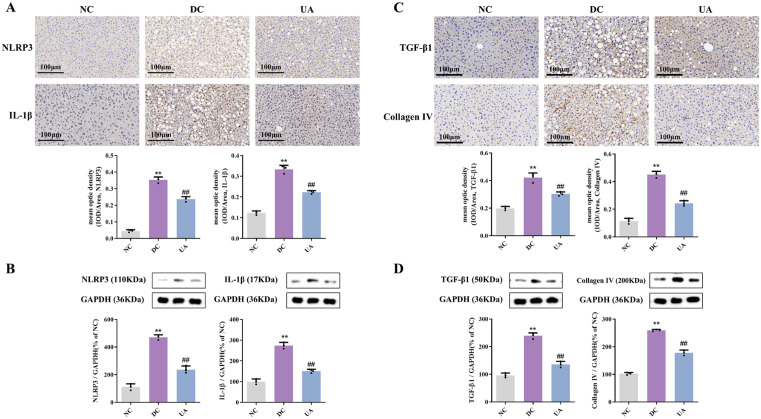
UA alleviates liver inflammation and fibrosis in T2DM mice. **(A)** Immunohistochemical images of NLRP3 and IL-1β (200×) **(B)** The expression of NLRP3 and IL-1β in liver of mice. **(C)** Immunohistochemical images of TGF-β1 and collagen IV. **(D)** The expression of TGF-β1 and collagen Ⅳ in liver of mice. ^*##*^*P* < 0.01 versus DC group; ^**^P < 0.01 versus NC group. The data were presented using means ± SD (n = 3).

The NLRP3 inflammasome and its regulated inflammatory factor IL-1β have been demonstrated to regulate the inflammatory response and can be used as an indicator to respond to liver inflammation in mice. Immunohistochemical analysis (Fig 5A) and Western blotting (Fig 5B) analysis demonstrated significant upregulation of NLRP3 inflammasome components and IL-1β in DC group hepatocytes compared to NC controls (*P* < 0.01), while UA treatment markedly suppressed their expression (*P* < 0.01). These findings suggest that UA has the capacity to downregulate the expression levels of NLRP3 and IL-1β proteins and to inhibit inflammation in the livers of T2DM mice.

Changes in the protein expression levels of TGF-β1 and collagen Ⅳ play an important role in the process of liver fibrosis, which can reflect collagen proliferation and the degree of fibrosis in liver tissue. The results of immunohistochemistry (Fig 5C) and Western blotting (Fig 5D) showed that the expression of TGF-β1 and collagen Ⅳ proteins increased significantly in the DC group compared with the NC group (*P* < 0.01), and the levels of both proteins in the livers of mice in the UA group were significantly reduced compared with those of mice in the DC group (*P* < 0.01). These findings suggest that UA has the ability to attenuate hepatic fibrosis in mice with T2DM.

### 3.5. UA ameliorated lipid accumulation and oxidative stress in LO2 Cells induced by HG combined with PA

**Fig 6:**
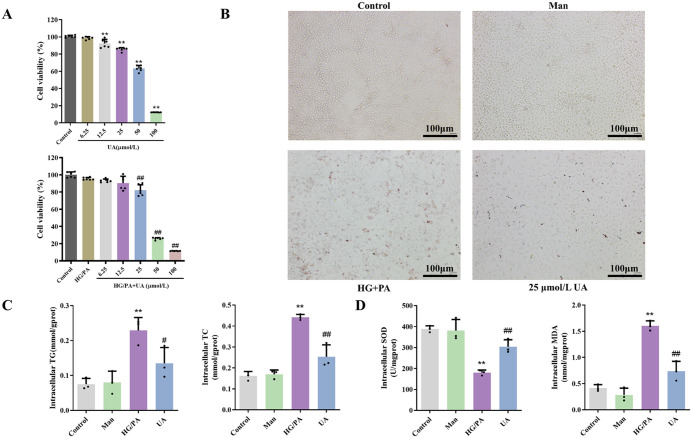
Effects of UA on oxidative stress and lipid accumulation in HG combined with PA-induced LO2 cells. **(A)** The viability of normal LO2 cells and HG combined with PA-induced LO2 cells following UA intervention was evaluated by MTT assay (n = 6). **(B)** LO2 cells, stained with Oil Red O, were observed in microscopy. **(C)** The content of TG and TC in LO2 cells. **(D)** The content of SOD and MDA in LO2 cells. ^*#*^*P* < 0.05, ^*##*^*P* < 0.01 versus HG/PA group; ^**^P < 0.01 versus Control group. The data were presented using means ± SD (n = 3).

To discuss the therapeutic effect of UA on cellular damage under hyperglycaemia and lipid deposition, a preliminary exploratory model was established by treating LO2 hepatocytes with a combination of high glucose and palmitic acid. The MTT assay results (Fig 6A) indicate a significant reduction in the survival viability of normal LO2 cells stimulated with 12.5, 25, 50, and 100 μmol/L UA. Similarly, the survival viability of LO2 cells induced by HG combined with PA was significantly reduced when the concentration of UA was up to 25, 50, and 100 μmol/L. According to the results, UA at a concentration of 25 μmol/L was selected for further experiments. The extent of lipid accumulation was determined by staining LO2 cells with Oil Red O. The results (Fig 6B) demonstrated that the deposition of lipids in LO2 cells, induced by the combination of HG and PA, was significantly ameliorated by UA. Furthermore, the contents of TG and TC in the LO2 cells of the UA group were found to be markedly reduced in comparison to those of the model group suggesting that UA had a modulating effect on the lipid levels of HG combined with PA-induced LO2 cells (Fig 6C). Meanwhile, the intervention of UA significantly increased the activity of SOD and led to a significant reduction in MDA. This suggests that UA reduces the level of oxidative stress in HG combined with PA-induced LO2 cells (Fig 6D).

### 3.6. UA reduces inflammation and fibrosis in HG combined with PA-induced LO2 cells

**Fig 7:**
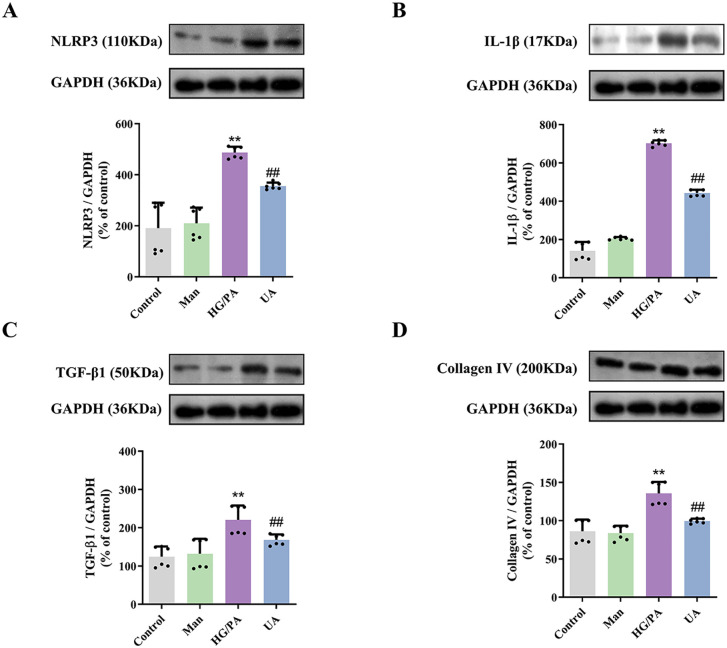
Effects of UA on the expression of inflammation and fibrosis-related proteins in HG combined with PA-induced LO2 cells. Representative images of western blotting detection of NLRP3 **(A)**, IL-1β **(B)**, TGF-β1 **(C)**, and Collagen IV **(D)**. ^**^P < 0.01 versus Control; ^*##*^*P* < 0.01 versus PA group. The data were presented using means ± SD (n = 6).

The results of the Western blotting indicate a substantial increase in the levels of all four proteins, namely NLRP3, IL-1β, TGF-β1, and collagen Ⅳ, in LO2 cells after the intervention of HG combined with PA. Conversely, the intervention of UA was observed to result in a notable reduction in the expression levels of NLRP3 and IL-1β in LO2 cells (Fig 7A and 7B). These findings indicate that UA may be an effective method for reducing the inflammation level of HG combined with PA-induced LO2 cells. The levels of TGF-β1 and collagen IV in LO2 cells induced by HG combined with PA were markedly reduced after handling with UA (Fig 7C and 7D). This suggests that UA has a fibrosis-reducing effect on LO2 cells induced by HG combined with PA.

### 3.7. NSS reversed the effect of UA on the inflammation in HG combined with PA-induced LO2 cells

**Fig 8:**
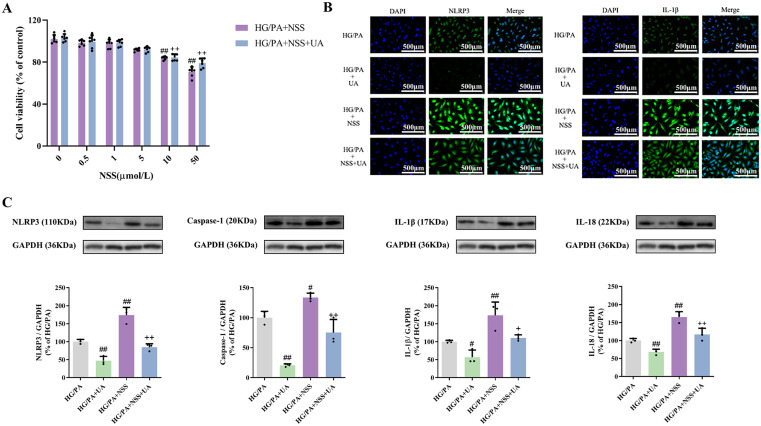
The reversal effects of NSS on UA. **(A)** Effects of different concentrations of NSS on the viability of LO2 cells induced by HG combined with PA (n = 6). **(B)** The expression of NLRP3 and IL-1 in LO2 cells was observed by immunofluorescence. **(C)** The expression of NLRP3, caspase-1, IL-1β and IL-18 in LO2 cells (n = 3). ^*#*^*P* < 0.05, ^*##*^*P* < 0.01 versus HG/PA group; ^*+*^*P* < 0.05, ^*++*^*P* < 0.01 versus HG/PA + NSS group. The data were presented using means ± SD.

To explore the correlation between the protective effect of UA on LO2 cells and the NLRP3-mediated signaling pathway, the NLRP3 agonist, NSS, was administered to LO2 cells. The results of MTT assay showed (Fig 8A) that there was no significant change in the survival viability of normal LO2 cells and HG combined with PA-intervened LO2 cells when the NSS concentrations were 0.5 μmol/L, 1 μmol/L, and 5 μmol/L. However, the survival viability of LO2 cells significantly decreased when stimulated with 10 μmol/L and 50 μmol/L concentration of NSS. For subsequent experiments, a concentration of 10 μmol/L NSS was chosen.

Immunofluorescence was used to detect NLRP3 and IL-1β expression in LO2 cells. The results showed that when NSS was given as an intervention, the fluorescence expression of NLRP3 and IL-1β in HG combined with PA-induced LO2 cells was markedly stronger than that in cells of the model group. In contrast, green fluorescence was significantly weakened compared to that of the NSS group (Fig 8B). Additionally, the green fluorescence in the cells of the NSS + UA group was significantly weakened compared to the NSS group.

Western blotting was performed to determine the expression of NLRP3, caspase-1, IL-1β, and IL-18 in LO2 cells. The results (Fig 8C) demonstrated that NSS reversed the effects of the UA, leading to a significant increase in the levels of NLRP3, caspase-1, IL-1β, and IL-18 in LO2 cells, which suggested that UA could protect against HG combined with PA-induced LO2 cell injury through the NLRP3 inflammasome signaling pathway.

## 4. Discussion

NAFLD has been recognized as the most common liver disease worldwide and one of the primary chronic complications of T2DM. Previous studies have demonstrated that diabetes is an independent risk factor for NAFLD progression [19], while NAFLD increases the incidence of T2DM and the risk of cardiovascular and renal complications [20]. Currently, there is a lack of effective clinical strategies for treating T2DM combined with NAFLD. Therefore, identifying pharmacological agents that concurrently offer hypoglycemic, lipid-regulating, and hepatoprotective effects is of great importance. Research by Tang et al. found that UA ameliorates diabetic conditions in high-fat diet-fed rats by modulating insulin signaling pathways [18]. Similarly, Kwon’s team reported that UA alleviates hepatic steatosis, fibrosis, and insulin resistance in mice induced by HFD, potentially through regulation of the circadian rhythm pathway [21]. Recent studies indicate that UA can mitigate cisplatin-induced hepatotoxicity through its antioxidant properties [22]. These findings suggest that UA demonstrates potential in improving glucose and lipid metabolism; however, they do not address whether UA ameliorates T2DM combined with NAFLD via the NLRP3 pathway. This gap underscores the need for further investigation in this direction.

Based on this, we established an in vivo model of T2DM combined with NAFLD in male C57BL/6J mice induced by HFD combined with STZ. Meanwhile, LO2 cells were treated with HG and PAto simulate cellular damage under hyperglycemic and lipid-overloaded conditions. This combination was specifically designed to recapitulate two core pathogenic drivers of diabetic NAFLD: chronic hyperglycemia and elevated circulating free fatty acids. 9 days after STZ injection, the FBG levels in the DC group mice exceeded or reached 11.1 mmol/L, indicating the successful establishment of a T2DM mouse model. According to our findings, UA significantly improved hepatic lipid deposition, reduced serum ALT and AST levels, and effectively alleviated oxidative stress, as evidenced by decreased MDA content and increased activity of SOD, CAT, and GSH-Px in liver tissue. These results indicate that UA exerts a protective effect on T2DM combined with NAFLD by improving oxidative stress.

Subsequently, we focused on hepatic inflammation and fibrosis to further investigate the mechanism of UA. In the progression of T2DM, chronic hyperglycemia, hyperlipidemia, and impaired insulin signaling collectively promote intracellular overproduction of ROS [23]. ROS serves as a key activator of the NLRP3 inflammasome—a multiprotein complex comprising the NLRP3 receptor, the adaptor protein ASC, and the effector caspase-1 [8]. Upon activation, NLRP3 recruits ASC and activates caspase-1, which cleaves pro-IL-1β into active IL-1β, thereby driving inflammatory responses [24]. Extensive literature indicates that inhibiting the maturation and release of IL-1β/IL-18 represents a core mechanism for blocking the inflammasome pathway and achieving significant anti-inflammatory effects [25,26]. In studies related to diabetes and metabolic liver injury, the upregulation of NLRP3 protein levels has been demonstrated to be functionally linked to inflammasome assembly and caspase-1 activation [27,28]. Simultaneously, excessive ROS further activates hepatic macrophages and HSCs, which in turn generate more ROS through pathways such as NADPH oxidase and release pro-inflammatory factors including TNF-α, IL-1β, and TGF-β. These inflammatory signals create a feedback loop that exacerbates oxidative stress and promotes the transformation of HSCs into collagen-producing myofibroblasts, collectively contributing to the persistence of hepatic inflammation and the progression of fibrosis [29]. Therefore, we hypothesized that the NLRP3 signaling pathway contributes to the protective effects of UA against inflammation and fibrosis in NAFLD. To validate this hypothesis, the NLRP3 agonist Nigericin sodium salt (NSS) was employed. As anticipated, UA inhibited the activation of the NLRP3 inflammasome, significantly down-regulated the expression levels of the pro‑inflammatory factors IL‑1β and IL‑18 as well as the pro‑fibrotic factor TGF‑β1, while also reducing the deposition of type IV collagen. This finding provides circumstantial evidence that UA suppresses the assembly and activation of the NLRP3 inflammasome as a key mechanism. Collectively, our data demonstrate that UA ameliorates T2DM combined with NAFLD by regulating the NLRP3 signaling pathway, thereby attenuating inflammatory responses and exerting anti-fibrotic effects.

Our experimental results demonstrated that UA intervention significantly reversed the elevated expression of TG, TC, ALT, and AST, ameliorating liver injury and lipid deposition. Meanwhile, UA treatment markedly enhanced the activities of antioxidant enzymes (SOD, CAT, and GSH-Px) in the livers of diabetic mice and in injured LO2 cells, while reducing the content of the lipid peroxidation product MDA. Further mechanistic investigation revealed that UA significantly downregulated the expression of NLRP3, IL-1β, TGF-β1, and collagen IV in the liver, suggesting that UA may alleviate oxidative stress, inflammation, and fibrosis through modulation of the NLRP3 pathway. This study provides experimental evidence supporting the potential of UA in treating T2DM-associated NAFLD. However, several key issues need to be addressed for its clinical application. The problem of poor oral bioavailability of ursolic acid remains unsolved, which directly affects its therapeutic efficacy. Future studies should focus on optimizing formulations to improve solubility and hepatic targeting, as well as combination therapies (such as with GLP-1 receptor agonists) to enhance efficacy.

Nevertheless, this study is subject to certain limitations. First, as an immortalized cell line, LO2 hepatocytes may not fully reflect the physiology of primary hepatocytes under diabetic conditions. Second, a lack of quantitative data limits the objective evaluation of both animal phenotypes and histopathological changes. Furthermore, while our findings suggest that UA alleviates liver injury via NLRP3 inflammasome inhibition, more direct mechanistic evidence—such as the use of NLRP3‑knockout models or specific NLRP3 pharmacological inhibitors—is required to definitively establish this causal relationship. To fully elucidate the therapeutic and safety profile of UA, future studies should employ more physiologically relevant models and utilize genetic or pharmacological approaches to directly validate the role of NLRP3 in its mechanism of protection.

## 5. Conclusions

In summary, UA can alleviate hepatic lipid deposition, resist oxidative stress, inhibit inflammation and fibrosis processes, and improve liver injury. The protective effect of UA on T2DM complicated with NAFLD may be related to the regulation of the NLRP3 inflammasome signaling pathway and the inhibition of downstream inflammatory factors. This study provides an idea for the treatment of T2DM with NAFLD. However, further elucidation is required regarding the mechanism of UA and its druggability.

## Supporting information

S1 File Raw images. (PDF)

S2 File Original data. (XLSX)
