# Supplementary material for: Ursolic acid alleviates liver injury in diabetic mice induced by high-fat diet combined with streptozotocin via the NLRP3 signaling pathway
Source: PLoS One. 2026 Feb 4;21(2):e0340643. doi: 10.1371/journal.pone.0340643 (PMC12871989; doi:10.1371/journal.pone.0340643)

The panel represents western blot analysis of NLRP3(110kDa) shown in Fig 5B.

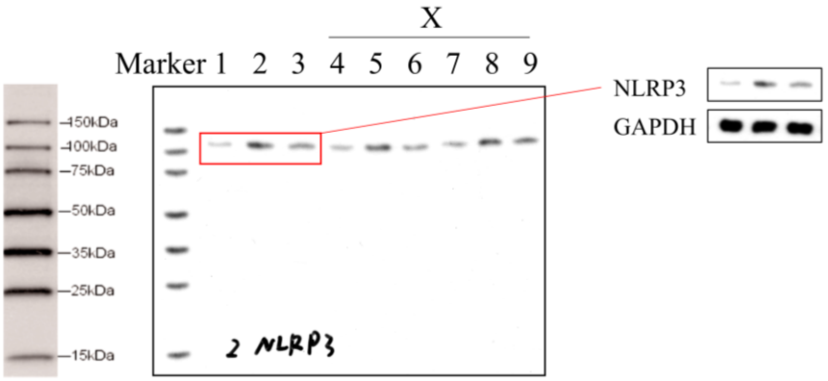

The panel represents western blot analysis of IL-1β(17kDa) shown in Fig 5B.

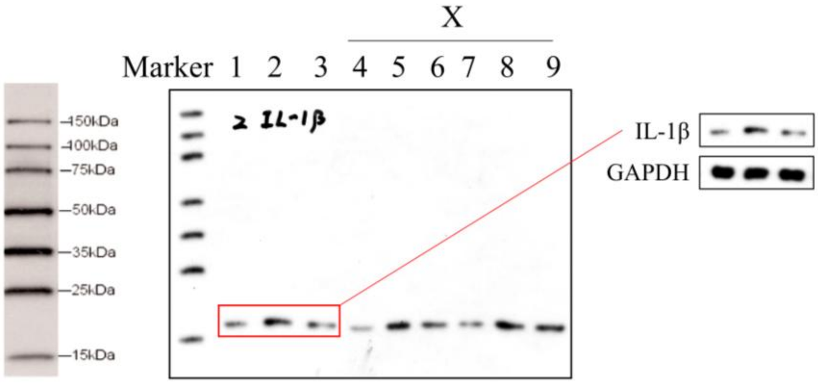

The panel represents western blot analysis of GAPDH(36kDa) shown in Fig 5B.

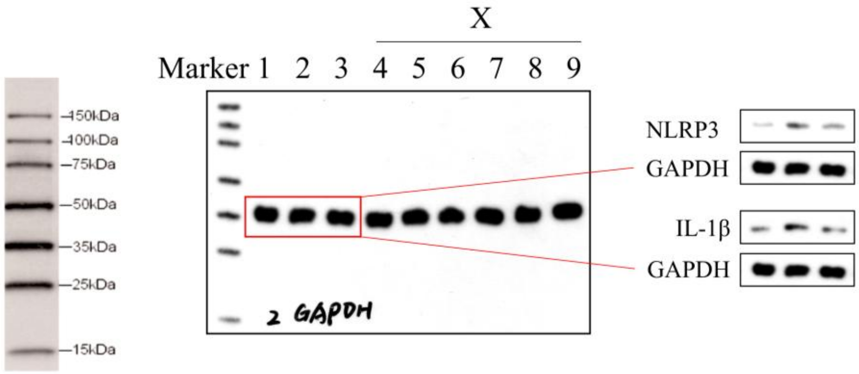

The panel represents western blot analysis of TGF-β1(50;12.5kDa) shown in Fig 5D.

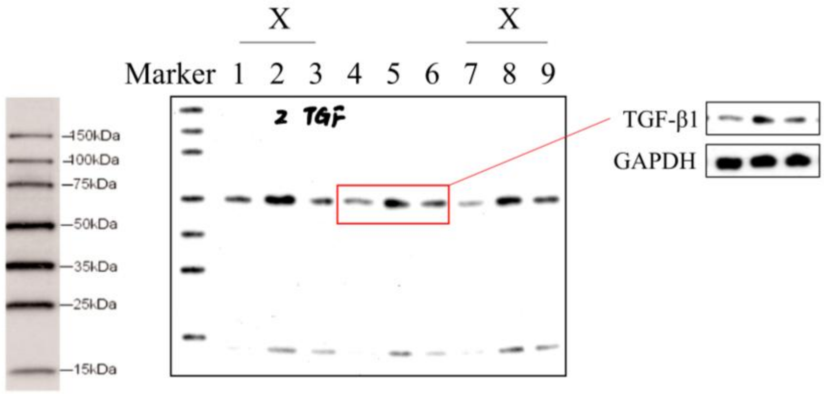

The panel represents western blot analysis of Collagen IV(200kDa) shown in Fig 5D.

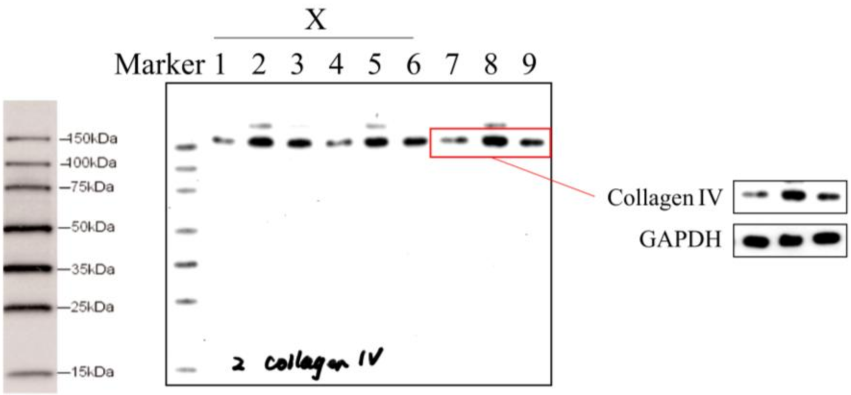

The panel represents western blot analysis of GAPDH(36kDa) shown in Fig 5D.

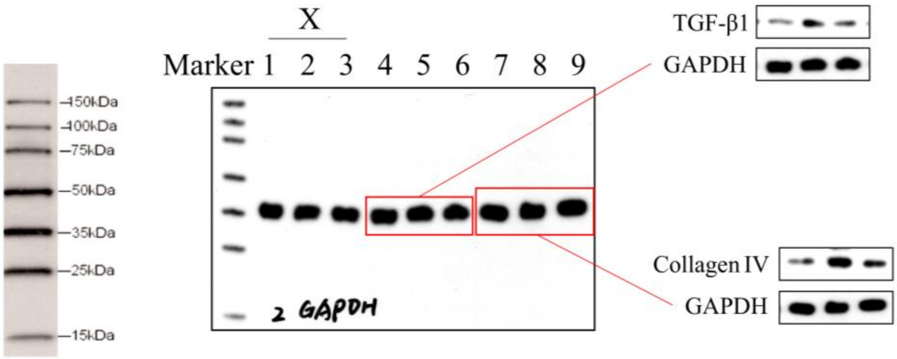

The panel represents western blot analysis of NLRP3(110kDa) shown in Fig 7A.

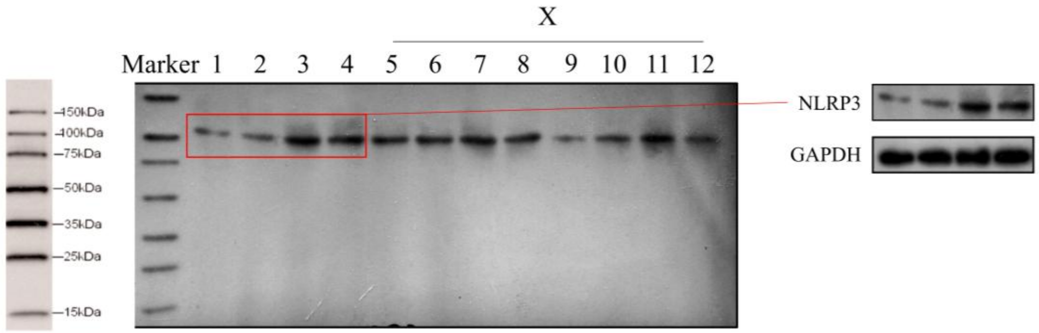

The panel represents western blot analysis of IL-1β(31;17kDa) shown in Fig 7B.

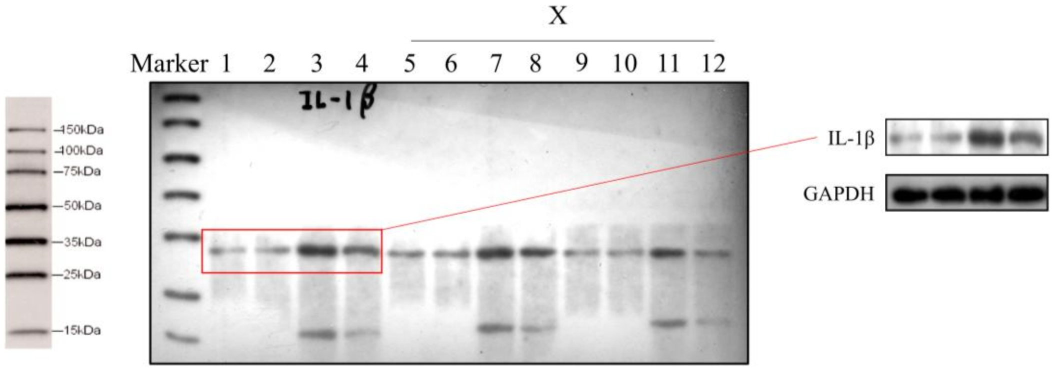

The panel represents western blot analysis of TGF-β1(50;12.5kDa) shown in Fig 7C.

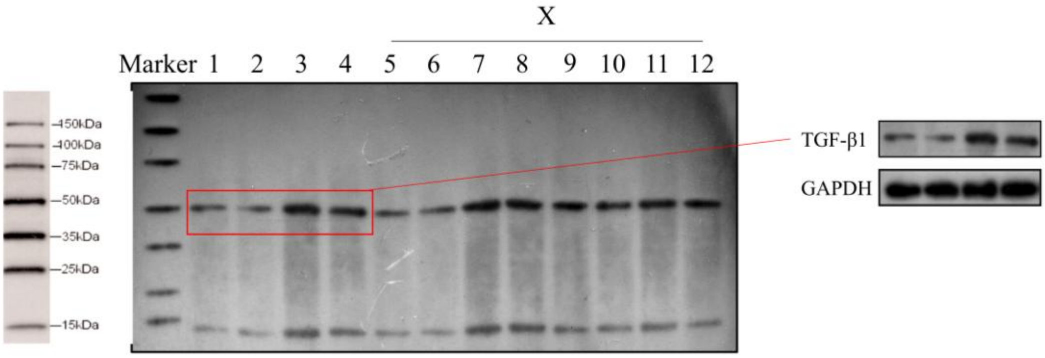

The panel represents western blot analysis of Collagen IV(200kDa) shown in Fig 7D.

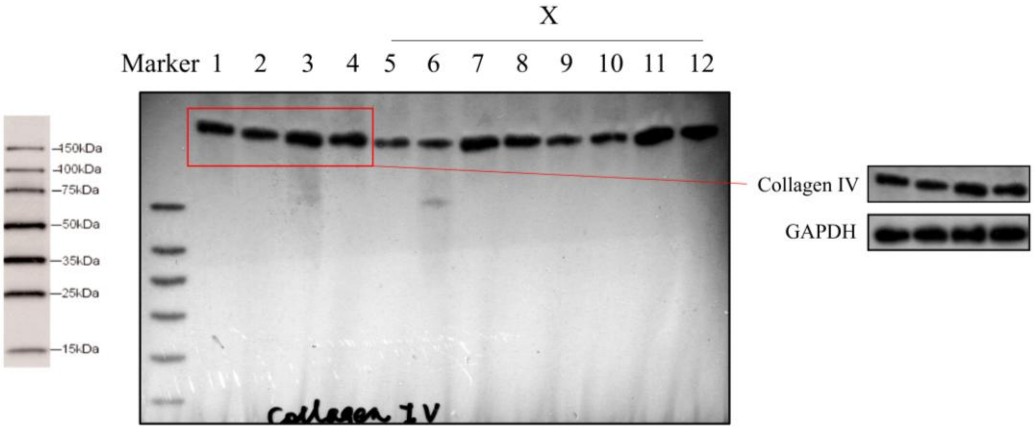

The panel represents western blot analysis of GAPDH(36kDa) shown in Fig 7.

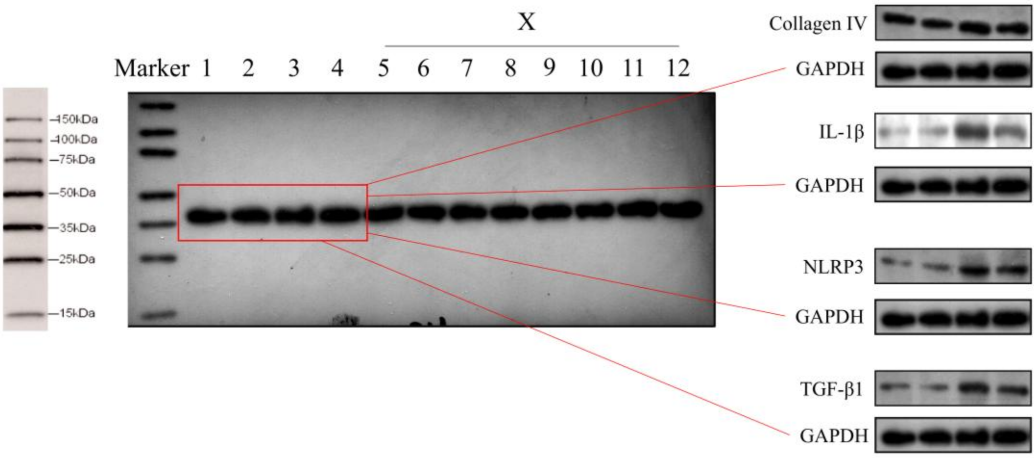

The panel represents western blot analysis of NLRP3 (110kDa) shown in Fig 8C.

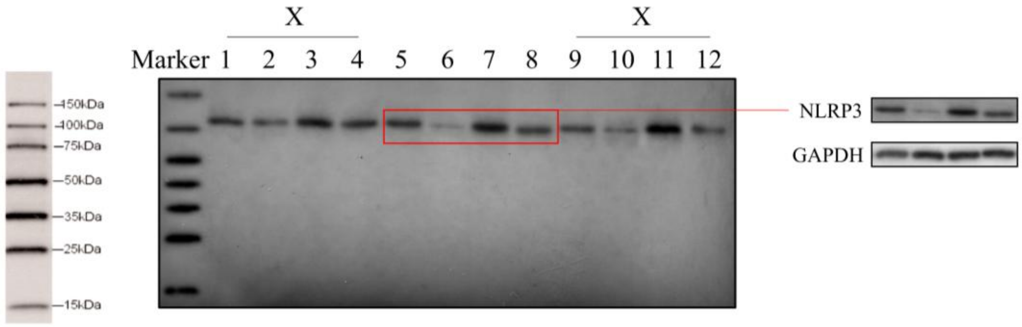

The panel represents western blot analysis of Caspase-1 (22;20kDa) shown in Fig 8C.

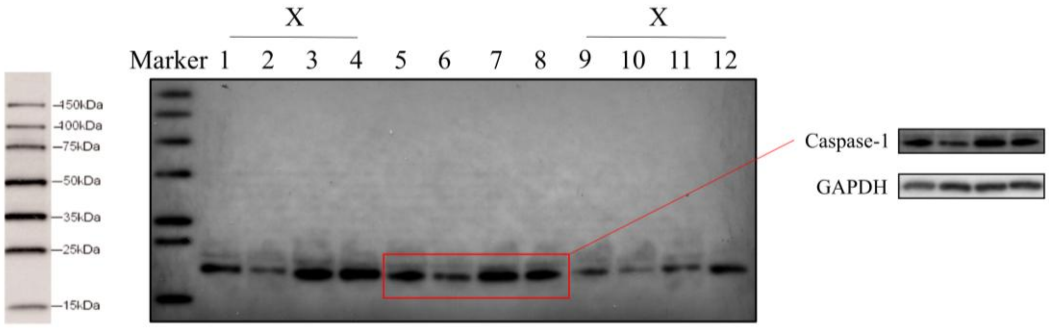

The panel represents western blot analysis of IL-1β(31;17kDa) shown in Fig 8C.

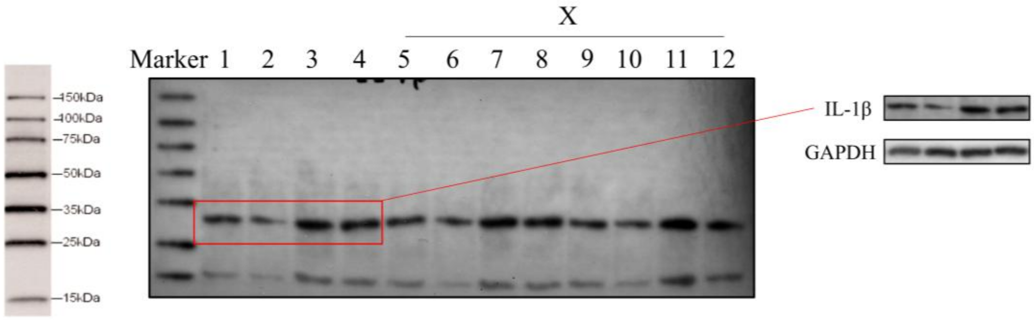

The panel represents western blot analysis of IL-18 (22kDa) shown in Fig 8C.

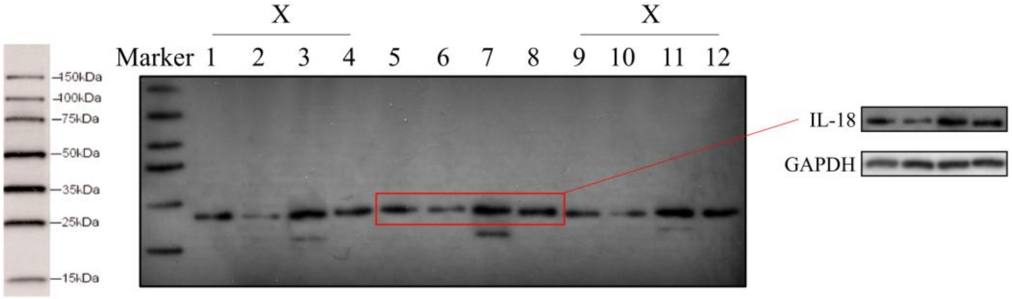

The panel represents western blot analysis of GAPDH (36kDa) shown in Fig 8C.

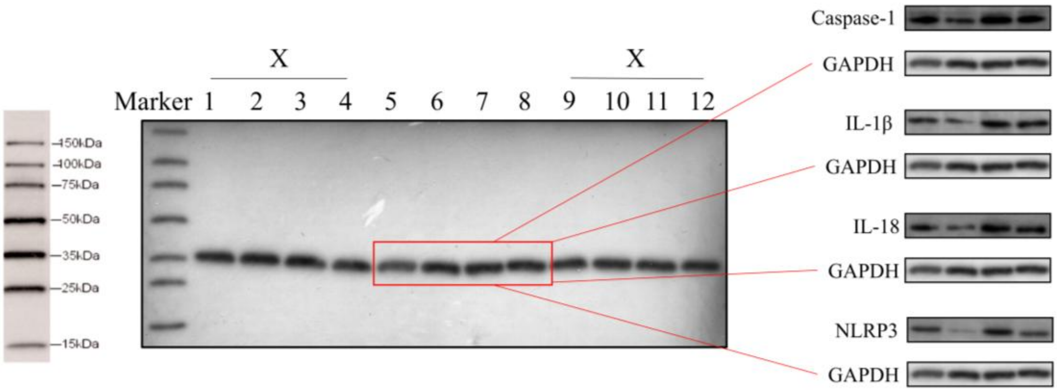

Supplement: S1 File — (PDF) [file pone.0340643.s001.pdf]
